# Supplementary material for: Redirecting antibody responses from egg-adapted epitopes following repeat vaccination with recombinant or cell culture-based versus egg-based influenza vaccines
Source: Nat Commun. 2024 Jan 4;15:254. doi: 10.1038/s41467-023-44551-x (PMC10767121; doi:10.1038/s41467-023-44551-x)
Supplement: Supplementary file 1 — Supplementary Information [file 41467_2023_44551_MOESM1_ESM.pdf]

Supplementary Materials

Redirecting antibody responses from egg-adapted epitopes following repeat vaccination with recombinant or cell culture-based versus egg-based influenza vaccines

Feng Liu,[1] F Liaini Gross,[1] Sneha Joshi,[1] Manjusha Gaglani,[2,3,4] Allison L. Naleway,[5] Kempapura Murthy,[4] Holly C. Groom,[5] Meredith G. Wesley,[1,6] Laura J. Edwards,[6] Lauren Grant,[1] Sara S. Kim,[1] Suryaprakash Sambhara,[1] Shivaprakash Gangappa,[1] Terrence Tumpey,[1] Mark G. Thompson[1], Alicia M. Fry,[1] Brendan Flannery,[1] Fatimah S. Dawood,[1] and Min Z Levine[1]*

[1]Influenza Division, Centers for Disease Control and Prevention, Atlanta, GA, USA

[2] Baylor Scott & White Health, Temple, TX, USA.

[3]Baylor College of Medicine, Temple, TX, USA

[4] Texas A & M University, College of Medicine, Temple, TX, USA

[5] Kaiser Permanente Northwest Center for Health Research, Portland, OR, USA

[6] Abt Associates, Atlanta, GA, USA

***Corresponding Author:** Min Z Levine, Ph. D., Influenza Division, Centers for Disease Control and Prevention. 1600 Clifton Road, Atlanta, Georgia 30329. mlevine@cdc.gov, Tel: 404-639-3504

Keywords: Antibody response, vaccine egg-adaption, egg-based, cell-based, recombinant quadrivalent influenza vaccines, healthcare personnel

Table S1. Proportions of MN or HI titers ≥ 40 (seropositivity rates) in year 1.

Vaccine groups	Age groups (yrs)	Time points	N	A(H3N2)		A(H1N1)pdm09		B/VIC		B/YAM	
				egg virus	cell virus	egg virus	cell virus	egg virus	cell virus	egg virus	cell virus
				%, 95% CI	%, 95% CI	%, 95% CI	%, 95% CI	%, 95% CI	%, 95% CI	%, 95% CI	%, 95% CI
Fluzone IIV4	18-44	Day 0	52	96 (91-102)	90 (91-102)	71 (58-84)	71 (58-84)	85 (74-95)	63 (74-95)	81 (70-92)	77 (70-92)
		1 Month	52	100 (-)	94 (-)	85 (74-95)	83 (74-95)	94 (88-101)	77 (88-101)	88 (79-97)	77 (79-97)
		6 Month	47	100 (-)	89 (-)	78 (66-91)	70 (66-91)	91 (83-100)	65 (83-100)	80 (69-92)	65 (69-92)
	45-64	Day 0	58	98 (95-102)	81 (95-102)	53 (40-67)	53 (40-67)	86 (77-95)	71 (77-95)	74 (63-86)	66 (63-86)
		1 Month	58	100 (-)	86 (-)	66 (53-78)	60 (53-78)	97 (92-101)	76 (92-101)	86 (77-95)	71 (77-95)
		6 Month	54	100 (-)	71 (-)	41 (27-55)	43 (27-55)	84 (74-95)	71 (74-95)	67 (53-80)	59 (53-80)
Fluarix IIV4	18-44	Day 0	55	100 (-)	89 (-)	75 (63-86)	75 (63-86)	98 (95-102)	84 (95-102)	87 (78-96)	84 (78-96)
		1 Month	55	100 (-)	87 (-)	89 (81-98)	84 (81-98)	96 (91-101)	95 (91-101)	95 (88-101)	95 (88-101)
		6 Month	48	98 (94-102)	77 (94-102)	77 (64-89)	79 (64-89)	94 (86-101)	77 (86-101)	81 (69-93)	81 (69-93)
	45-64	Day 0	65	97 (92-101)	73 (92-101)	50 (37-63)	50 (37-63)	94 (88-100)	73 (88-100)	91 (83-98)	73 (83-98)
		1 Month	63	98 (95-102)	79 (95-102)	78 (67-88)	78 (67-88)	98 (95-102)	90 (95-102)	92 (85-99)	83 (85-99)
		6 Month	59	95 (89-101)	64 (89-101)	56 (43-69)	58 (43-69)	97 (92-101)	76 (92-101)	86 (77-95)	75 (77-95)
ccIIV4	18-44	Day 0	135	99 (96-101)	78 (96-101)	64 (55-72)	64 (55-72)	93 (88-97)	73 (88-97)	89 (84-94)	79 (84-94)
		1 Month	133	99 (98-101)	85 (98-101)	82 (75-89)	81 (75-89)	95 (91-99)	80 (91-99)	92 (88-97)	86 (88-97)
		6 Month	124	97 (95-100)	74 (95-100)	76 (68-84)	72 (68-84)	85 (78-91)	66 (78-91)	86 (80-93)	71 (80-93)
	45-64	Day 0	148	95 (91-98)	76 (91-98)	55 (47-64)	55 (47-64)	95 (91-98)	76 (91-98)	86 (80-91)	68 (80-91)
		1 Month	147	96 (93-99)	83 (93-99)	73 (66-81)	71 (66-81)	97 (94-100)	90 (94-100)	89 (84-94)	78 (84-94)
		6 Month	142	92 (87-96)	73 (87-96)	72 (64-80)	69 (64-80)	93 (89-98)	81 (89-98)	85 (79-91)	66 (79-91)
RIV4	18-44	Day 0	99	100 (-)	88 (-)	74 (65-83)	74 (65-83)	88 (81-94)	71 (81-94)	90 (84-96)	83 (84-96)
		1 Month	98	100 (-)	96 (-)	86 (79-93)	91 (79-93)	97 (93-100)	80 (93-100)	98 (95-101)	96 (95-101)
		6 Month	85	100 (-)	95 (-)	83 (74-91)	88 (74-91)	89 (82-96)	70 (82-96)	95 (90-100)	86 (90-100)
	45-64	Day 0	98	95 (90-99)	79 (90-99)	60 (50-70)	60 (50-70)	90 (84-96)	74 (84-96)	76 (67-84)	60 (67-84)
		1 Month	98	100 (-)	100 (-)	79 (70-87)	78 (70-87)	95 (90-99)	86 (90-99)	88 (81-94)	80 (81-94)
		6 Month	92	99 (97-101)	94 (97-101)	54 (44-65)	52 (44-65)	93 (88-99)	81 (88-99)	81 (73-89)	76 (73-89)

% ≥ 40: proportions of participants with MN titer of 40 or greater to A(H3N2) virus, HI titer of 40 or greater to A(H1N1)pdm09 and B viruses respectively.

Table S2. Seroconversion rates at 1-month post-vaccination in year 1 participants.

Vaccine groups	Age groups (yrs)	N	A(H3N2)		A(H1N1)pdm09		B/VIC		B/YAM	
			Egg virus	Cell virus	Egg virus	Cell virus	Egg virus	Cell virus	Egg virus	Cell virus
			%, 95% CI	%, 95% CI	%, 95% CI	%, 95% CI	%, 95% CI	%, 95% CI	%, 95% CI	%, 95% CI
Fluzone IIV4	18-44	52	13 (4-23)	12 (3-21)	17 (7-28)	15 (5-26)	15 (5-26)	12 (3-21)	10 (1-18)	8 (0-15)
	45-64	58	21 (10-31)	9 (1-16)	9 (1-16)	5 (-1-11)	7 (0-14)	5 (-1-11)	12 (3-21)	7 (0-14)
Fluarix IIV4	18-44	55	2 (-2-5)	0 (-)	15 (5-24)	13 (4-22)	5 (-1-12)	5 (-1-12)	9 (1-17)	7 (0-14)
	45-64	63	19 (9-29)	6 (0-13)	27 (16-38)	27 (16-38)	17 (8-27)	8 (1-15)	19 (9-29)	11 (3-19)
ccIIV4	18-44	133	7 (2-11)	14 (8-20)	8 (4-13)	10 (5-15)	5 (1-9)	7 (2-11)	8 (4-13)	7 (2-11)
	45-64	147	9 (4-13)	13 (7-18)	17 (11-23)	16 (10-22)	2 (0-4)	5 (2-9)	7 (3-12)	7 (3-11)
RIV4	18-44	98	19 (11-27)	43 (33-53)	20 (12-29)	24 (16-33)	15 (8-23)	12 (6-19)	21 (13-30)	21 (13-30)
	45-64	98	42 (32-52)	56 (46-66)	26 (17-34)	27 (18-35)	10 (4-16)	11 (5-18)	19 (11-27)	13 (6-20)

SCR: seroconversion rate, defined as ≥ 4-fold rise of antibody titers from pre to 1-month post-vaccination with post-vaccination titer is ≥ 40.

Table S3. Vaccine breakthrough cases by vaccine groups and infection subtypes in year 1.

Vaccine groups	PCR confirmed infection			
	A(H3N2)	A(H1N1)pdm09	Influenza A, unsubtypable	Total
Fluzone IIV4	2	2	0	4
Fluarix IIV4	1	0	0	1
ccIIV4	9	4	1	14
RIV4	2	4	1	7
Total	14	10	2	26

Table S4. Proportions of HI titers ≥ 40 (seropositivity rates) in year 2 participants.

Vaccine groups	Age group (yrs)	N	Time points	A(H3N2)				A(H1N1)pdm09				B/YAM				B/VIC			
				egg virus		cell virus		egg virus		cell virus		egg virus		cell virus		egg virus		cell virus	
				%	95% CI	%	95% CI	%	95% CI	%	95% CI	%	95% CI	%	95% CI	%	95% CI	%	95% CI
ccIIV4-ccIIV4	18-44	42	Day 0	86	(74 - 96)	72	(58 - 86)	78	(66 - 92)	64	(50 - 80)	84	(72 - 96)	78	(66 - 92)	86	(74 - 96)	60	(44 - 76)
			1 month	100	(-)	96	(88 - 102)	86	(74 - 96)	76	(62 - 90)	92	(84 - 100)	90	(82 - 100)	98	(92 - 102)	72	(58 - 86)
	45-64	59	Day 0	84	(74 - 92)	68	(56 - 80)	58	(44 - 70)	32	(20 - 44)	78	(68 - 88)	64	(52 - 76)	84	(76 - 94)	58	(44 - 70)
			1 month	94	(90 - 100)	94	(86 - 100)	76	(66 - 88)	70	(58 - 82)	90	(82 - 98)	76	(66 - 88)	90	(82 - 98)	72	(62 - 84)
ccIIV4-RIV4	18-44	45	Day 0	78	(66 - 90)	76	(62 - 88)	64	(50 - 78)	46	(32 - 62)	78	(66 - 90)	68	(54 - 82)	88	(80 - 98)	64	(50 - 78)
			1 month	92	(82 - 100)	96	(90 - 102)	84	(74 - 96)	84	(74 - 96)	96	(90 - 102)	96	(90 - 102)	88	(80 - 98)	82	(70 - 94)
	45-64	61	Day 0	92	(84 - 98)	72	(60 - 84)	36	(24 - 48)	26	(14 - 38)	82	(72 - 92)	58	(44 - 70)	94	(88 - 100)	56	(42 - 68)
			1 month	96	(92 - 102)	96	(92 - 102)	74	(62 - 86)	70	(58 - 82)	92	(84 - 98)	86	(78 - 96)	98	(96 - 102)	84	(74 - 94)
RIV4-ccIIV4	18-44	28	Day 0	100	(-)	90	(78 - 102)	72	(54 - 90)	58	(38 - 76)	86	(72 - 100)	82	(68 - 98)	82	(68 - 98)	54	(34 - 74)
			1 month	100	(-)	96	(90 - 104)	96	(90 - 104)	92	(82 - 104)	90	(78 - 102)	86	(72 - 100)	90	(78 - 102)	64	(46 - 84)
	45-64	45	Day 0	96	(90 - 102)	92	(82 - 100)	54	(38 - 68)	44	(30 - 60)	86	(76 - 96)	72	(58 - 84)	86	(76 - 96)	68	(54 - 82)
			1 month	94	(86 - 100)	88	(80 - 98)	82	(70 - 94)	74	(60 - 86)	94	(86 - 100)	82	(70 - 94)	92	(82 - 100)	78	(66 - 90)
RIV4-RIV4	18-44	30	Day 0	86	(74 - 100)	84	(70 - 98)	86	(74 - 100)	70	(52 - 88)	94	(84 - 102)	84	(70 - 98)	96	(90 - 104)	74	(56 - 90)
			1 month	100	(-)	100	(-)	94	(84 - 102)	90	(78 - 102)	100	(-)	100	(-)	96	(90 - 104)	76	(60 - 92)
	45-64	44	Day 0	96	(90 - 102)	98	(94 - 102)	64	(48 - 78)	48	(32 - 64)	86	(76 - 96)	64	(48 - 78)	84	(72 - 96)	70	(56 - 84)
			1 month	100	(-)	100	(-)	82	(70 - 94)	70	(56 - 84)	94	(86 - 100)	82	(70 - 94)	94	(86 - 100)	88	(78 - 98)
IIV4-ccIIV4	18-44	49	Day 0	86	(76 - 96)	72	(58 - 84)	76	(64 - 88)	58	(42 - 72)	82	(70 - 92)	80	(68 - 92)	88	(78 - 98)	70	(56 - 82)
			1 month	92	(84 - 100)	90	(82 - 98)	92	(84 - 100)	76	(64 - 88)	90	(82 - 98)	82	(70 - 92)	98	(94 - 102)	74	(60 - 86)
	45-64	57	Day 0	68	(56 - 80)	56	(42 - 70)	52	(40 - 66)	36	(24 - 50)	72	(60 - 84)	48	(34 - 60)	92	(84 - 98)	54	(42 - 68)
			1 month	86	(76 - 96)	80	(70 - 92)	68	(56 - 80)	60	(46 - 72)	78	(68 - 90)	62	(48 - 74)	88	(78 - 96)	70	(58 - 82)
IIV4-RIV4	18-44	48	Day 0	86	(76 - 96)	80	(68 - 92)	86	(76 - 96)	68	(56 - 82)	86	(76 - 96)	82	(70 - 92)	88	(78 - 98)	68	(56 - 82)
			1 month	96	(90 - 102)	94	(86 - 100)	94	(86 - 100)	86	(76 - 96)	96	(90 - 102)	94	(86 - 100)	94	(86 - 100)	86	(76 - 96)
	45-64	56	Day 0	78	(68 - 90)	64	(52 - 78)	34	(22 - 46)	22	(10 - 32)	76	(66 - 88)	52	(38 - 66)	90	(80 - 98)	56	(42 - 68)
			1 month	94	(88 - 100)	94	(88 - 100)	62	(50 - 76)	60	(48 - 74)	88	(78 - 96)	88	(78 - 96)	98	(94 - 102)	82	(72 - 92)
IIV4-IIV4	18-44	47	Day 0	74	(62 - 88)	66	(52 - 80)	68	(54 - 82)	68	(54 - 82)	76	(64 - 90)	72	(60 - 86)	90	(80 - 98)	62	(48 - 76)
			1 month	94	(86 - 100)	92	(84 - 100)	72	(60 - 86)	68	(54 - 82)	94	(86 - 100)	82	(72 - 94)	98	(94 - 102)	74	(62 - 88)
	45-64	56	Day 0	78	(68 - 90)	78	(68 - 90)	48	(34 - 62)	34	(22 - 46)	76	(64 - 86)	54	(40 - 68)	82	(72 - 92)	42	(28 - 54)
			1 month	90	(80 - 98)	90	(80 - 98)	60	(48 - 74)	60	(48 - 74)	90	(80 - 98)	62	(50 - 76)	92	(86 - 100)	64	(52 - 78)

% ≥ 40: proportions of participants with HI titer of 40 or greater to A(H3N2), A(H1N1)pdm09 and B viruses respectively.

Table S5. Seroconversion rates at 1-month post-vaccination in year 2 participants.

Vaccine arm	Age group (yrs)	N	A(H3N2)				A(H1N1)pdm09				B/YAM				B/VIC			
			egg virus		cell virus		egg virus		cell virus		egg virus		cell virus		egg virus		cell virus	
			%,	95% CI	%,	95% CI	%,	95% CI	%,	95% CI	%,	95% CI	%,	95% CI	%,	95% CI	%,	95% CI
ccIIV4-ccIIV4	18-44	42	40	(24 - 56)	40	(24 - 56)	20	(6 - 32)	10	(0 - 18)	8	(0 - 16)	12	(2 - 22)	4	(-2 - 12)	8	(0 - 16)
	45-64	59	16	(8 - 26)	24	(12 - 34)	16	(8 - 26)	22	(12 - 32)	12	(4 - 20)	12	(4 - 20)	6	(0 - 10)	8	(2 - 16)
ccIIV4-RIV4	18-44	45	42	(28 - 58)	42	(28 - 58)	20	(8 - 32)	22	(10 - 34)	20	(8 - 32)	26	(14 - 40)	8	(0 - 18)	20	(8 - 32)
	45-64	61	26	(14 - 38)	48	(34 - 60)	38	(26 - 50)	44	(32 - 58)	26	(14 - 38)	22	(12 - 34)	10	(2 - 18)	16	(6 - 26)
RIV4-ccIIV4	18-44	28	14	(0 - 28)	8	(-4 - 18)	18	(2 - 32)	22	(6 - 38)	8	(-4 - 18)	4	(-4 - 10)	14	(0 - 28)	8	(-4 - 18)
	45-64	45	6	(0 - 14)	8	(0 - 18)	22	(10 - 34)	20	(8 - 32)	8	(0 - 18)	8	(0 - 18)	4	(-2 - 10)	6	(0 - 14)
RIV4-RIV4	18-44	30	44	(24 - 62)	44	(24 - 62)	10	(-2 - 22)	6	(-2 - 16)	14	(0 - 26)	20	(4 - 36)	4	(-4 - 10)	4	(-4 - 10)
	45-64	44	30	(16 - 44)	16	(4 - 28)	18	(6 - 30)	28	(14 - 40)	18	(6 - 30)	20	(8 - 32)	14	(4 - 24)	14	(4 - 24)
IIV4-ccIIV4	18-44	49	20	(8 - 32)	22	(10 - 34)	18	(8 - 30)	20	(8 - 32)	4	(-2 - 10)	6	(0 - 14)	2	(-2 - 6)	2	(-2 - 6)
	45-64	57	22	(10 - 32)	26	(14 - 38)	16	(6 - 26)	22	(10 - 32)	8	(2 - 16)	12	(4 - 22)	2	(-2 - 6)	8	(0 - 14)
IIV4-RIV4	18-44	48	34	(20 - 48)	44	(30 - 58)	22	(10 - 36)	20	(8 - 32)	18	(8 - 30)	20	(8 - 32)	20	(8 - 32)	22	(10 - 36)
	45-64	56	34	(22 - 46)	38	(24 - 50)	28	(16 - 40)	32	(20 - 44)	28	(16 - 40)	38	(24 - 50)	24	(12 - 34)	24	(12 - 34)
IIV4-IIV4	18-44	47	32	(18 - 46)	28	(14 - 40)	6	(0 - 14)	2	(-2 - 6)	4	(-2 - 10)	4	(-2 - 10)	10	(2 - 20)	2	(-2 - 6)
	45-64	56	20	(8 - 30)	18	(8 - 28)	8	(0 - 14)	8	(0 - 14)	12	(4 - 22)	2	(-2 - 6)	10	(2 - 20)	6	(0 - 12)

SCR: seroconversion rate, which is defined by ≥ 4-fold rise of antibody titers from pre to 1-month post-vaccination with post-vaccination titer is ≥ 40.

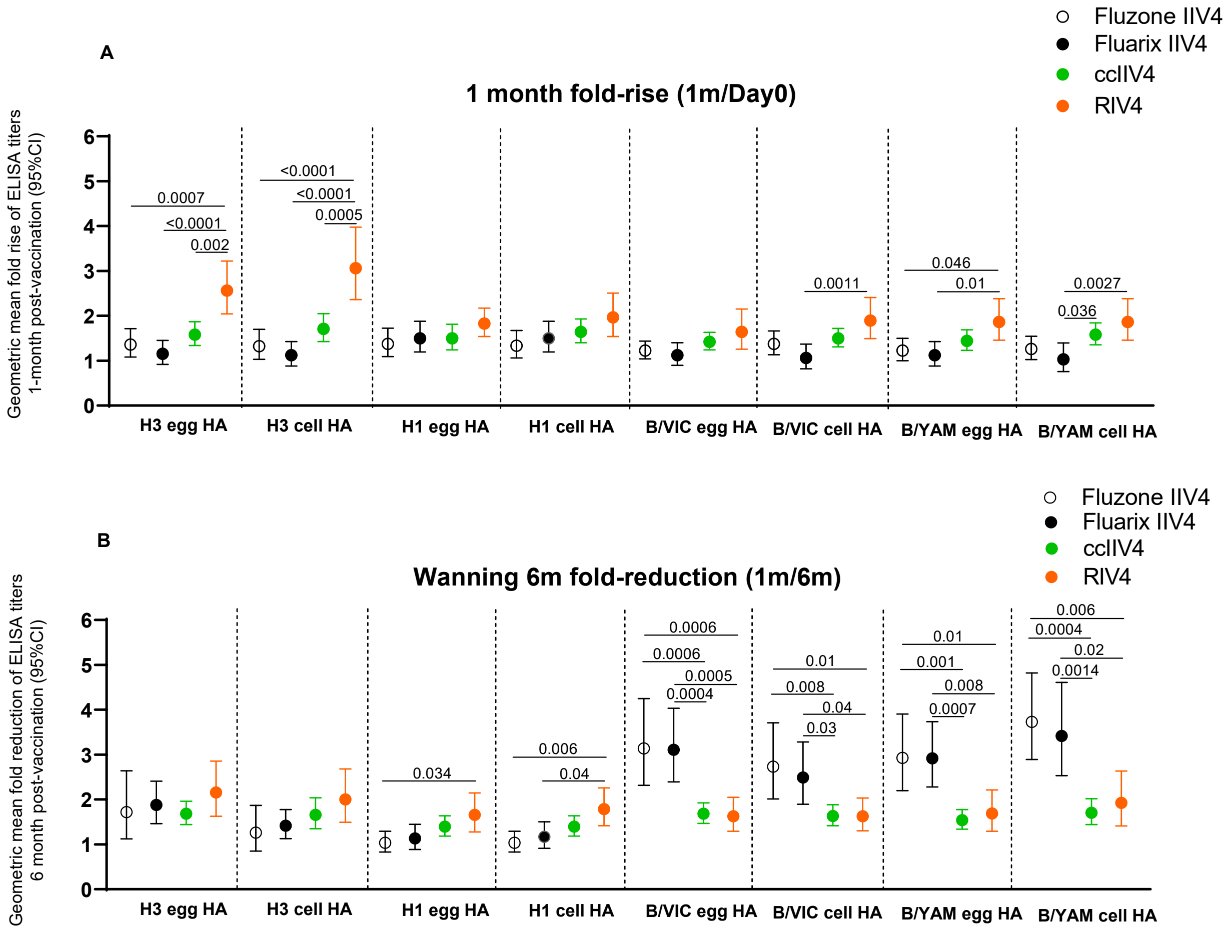

Figure S1. Fold rise of total HA binding antibody titers 1-month post-vaccination and waning of total binding antibody titers 6-month post-vaccination in year 1. A. Geometric mean fold-rise of ELISA antibody titers (1m/Day 0) from each of the four vaccine groups (combined 18-44 and 45-64 years age groups) were calculated for egg- and cell-vaccine virus HA respectively. Fluzone IIV4 (n=24), Fluarix IIV4 (n=24), ccIIV4 (n=54), RIV4 (n=40)). **B.** Antibody waning was expressed as the geometric mean fold-reduction of ELISA titers from 1-month to 6-month (1m/6m) post-vaccination. Fluzone IIV4 (n=21), Fluarix IIV4 (n=23), ccIIV4 (n=51), RIV4 (n=38)). One-way ANOVA corrected for multiple comparisons (Tukey's test) was used to compare the GMTs of each time point among the 4 vaccine groups. Statistically significant differences between groups are indicated by *p* values on the horizontal bars.

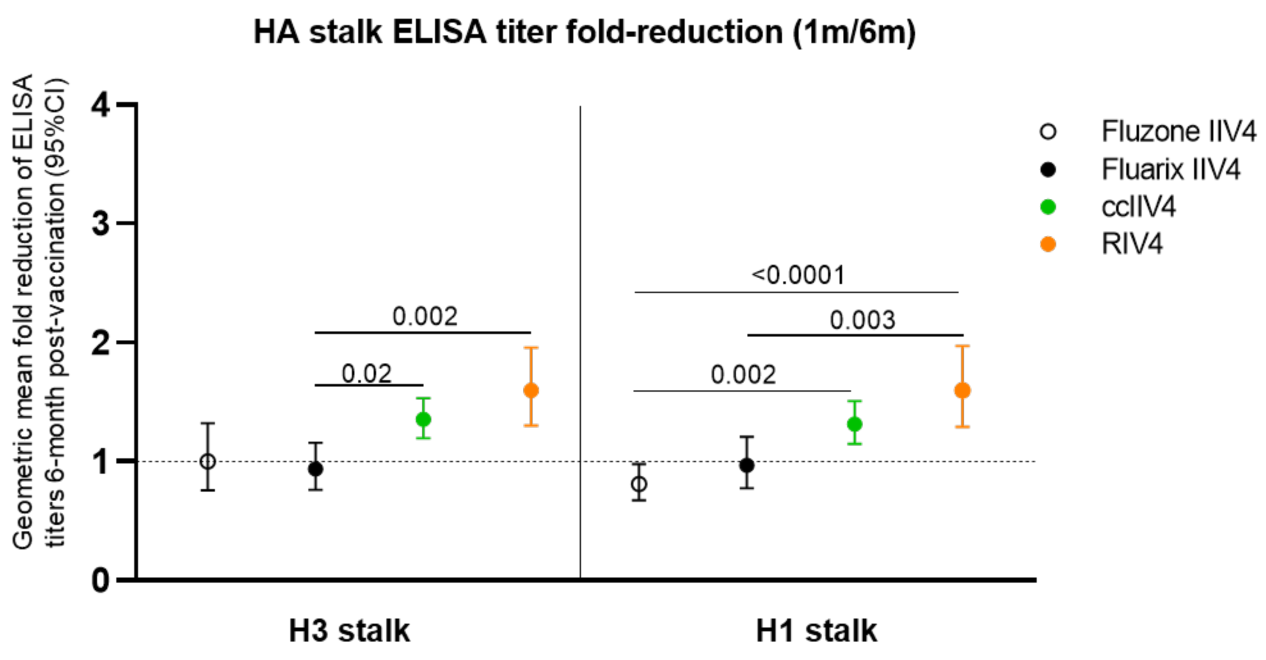

Figure S2. Waning of HA stalk antibodies comparing 1 month vs 6 months post-vaccination in Year 1. HA stalk antibody waning was analyzed as the fold-reduction of titers from 1-month to 6-month (1m/6m) post-vaccination. Geometric mean fold-reduction with 95% confidence interval (CI) was presented for each of the four vaccine groups (combined 18-44 and 45-64 years age groups) and for A(H3N2) and A(H1N1)pdm09 HA stalk respectively. Fluzone IIV4 (n=20), Fluarix IIV4 (n=22), ccIIV4 (n=48), RIV4 (n=37). One-way ANOVA nonparametric Kruskal-Wallis test was used to compare the GMTs of each time point among the 4 vaccine groups. Statistically significant differences between groups are indicated by *p* values on the horizontal bars.

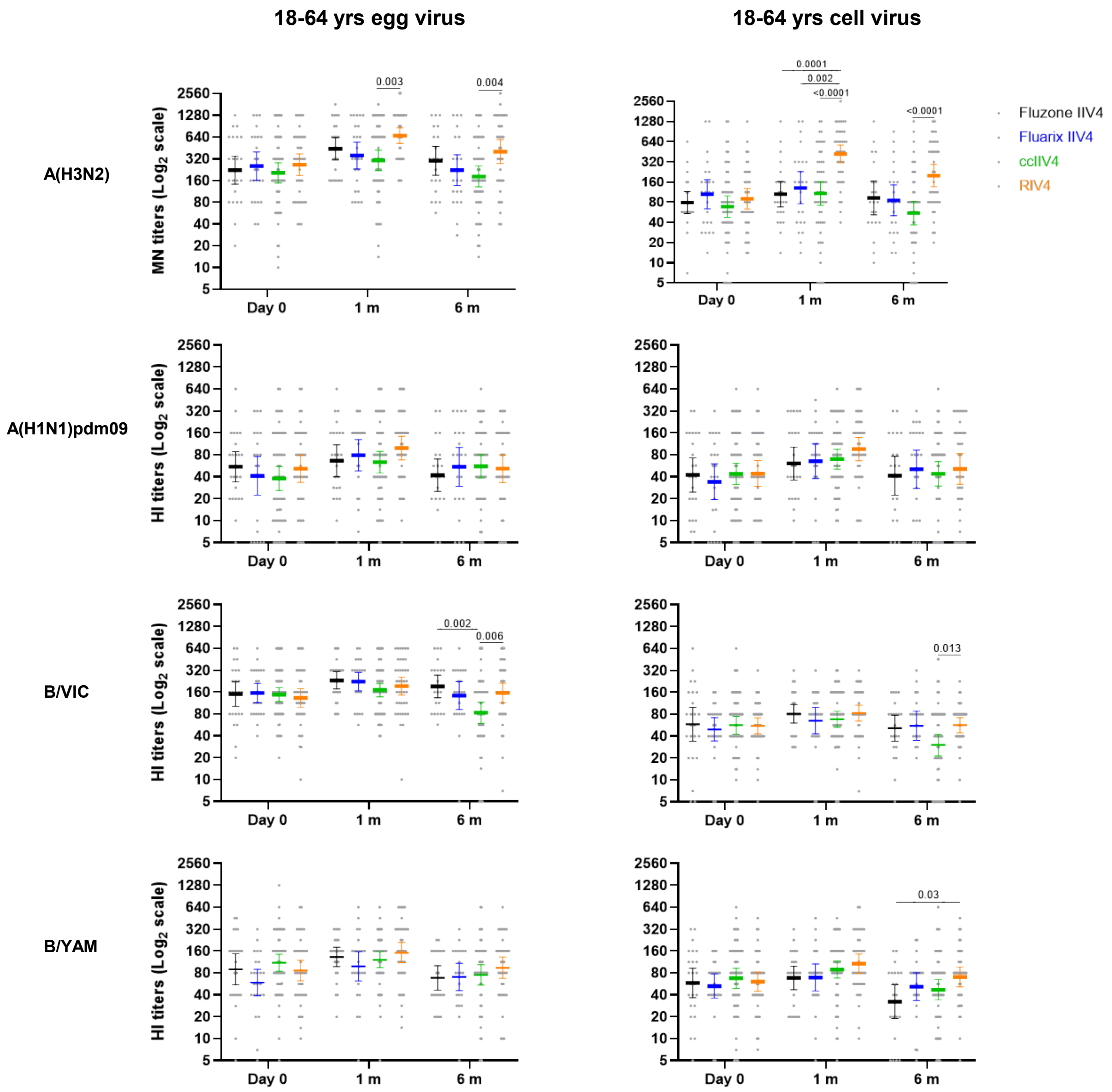

Figure S3. MN or HI antibody responses to egg- and cell-propagated vaccine viruses at pre-vaccination (Day 0), 1-month, and 6-month post-vaccination in year 1 participants selected for ELISA. Antibody titers from each of the four vaccine groups were presented as geometric mean titers (GMTs) with 95% confidence interval (CI) for combined 18-44 years and 45-64 years age groups. Grey dots represented individual titers. Fluzone IIV4: Day 0 (n=24), 1m (n=24), 6m (n=21); Fluarix IIV4: Day 0 (n=24), 1m (n=24), 6m (n=23); ccIIV4: Day 0 (n=56), 1m (n=54), 6m (n=51); RIV4: Day 0 (n=40), 1m (n=40), 6m (n=38). Microneutralization titers (MN) were analyzed for A(H3N2) virus, while hemagglutination inhibition (HI) titers were analyzed for A(H1N1)pdm09, B/Victoria (B/VIC), and B/Yamagata (B/YAM) viruses. One-way ANOVA corrected for multiple comparisons (Tukey's test) was used to compare the GMTs of each time point among the 4 vaccine groups. Statistically significant differences between groups are indicated by p values on the horizontal bars.

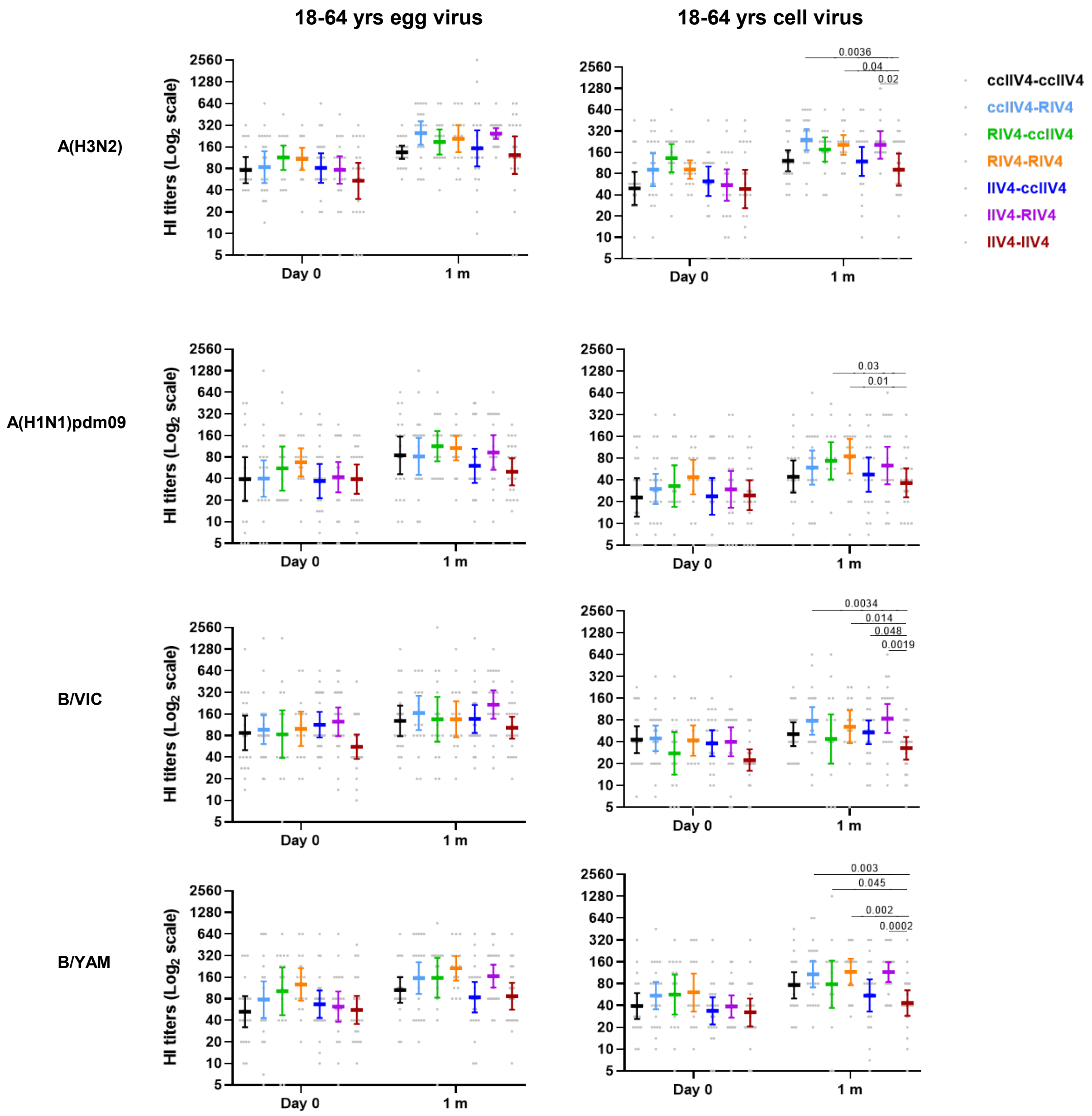

Figure S4. HI antibody responses to egg- and cell-propagated vaccine viruses at pre-vaccination (Day 0) and 1-month post-vaccination (1m) in year 2 participants selected for ELISA. Antibody titers from each of the 7 vaccine arms were presented as geometric mean titers (GMTs) with 95% confidence interval (CI) for combined 18-44 years and 45-64 years age groups. Grey dots represented individual titers. ccIIV4-ccIIV4 (n=20), ccIIV4-RIV4 (n=22), RIV4-ccIIV4 (n=16), RIV4-RIV4 (n=16), IIV4-ccIIV4 (n=22), IIV4-RIV4 (n=22), IIV4-IIV4 (n=22). Unpaired *t* test (two-tailed) was used to compare the GMT to cell vaccine virus between IIV4-IIV4 arm and each of the remaining 6 arms respectively. Statistically significant differences between groups are indicated by p values on the horizontal bars.
