# Supplementary material for: Redirecting antibody responses from egg-adapted epitopes following repeat vaccination with recombinant or cell culture-based versus egg-based influenza vaccines
Source: Nat Commun. 2024 Jan 4;15:254. doi: 10.1038/s41467-023-44551-x (PMC10767121; doi:10.1038/s41467-023-44551-x)
Supplement: Supplementary file 3 — Reporting Summary [file 41467_2023_44551_MOESM3_ESM.pdf]

nature portfolio

Corresponding author(s): Min Z Levine, PhD

Last updated by author(s): Dec 1, 2023

Reporting Summary

Nature Portfolio wishes to improve the reproducibility of the work that we publish. This form provides structure for consistency and transparency in reporting. For further information on Nature Portfolio policies, see our Editorial Policies and the Editorial Policy Checklist.

Statistics

For all statistical analyses, confirm that the following items are present in the figure legend, table legend, main text, or Methods section.

n/a	Confirmed	
☐	☒	The exact sample size (*n*) for each experimental group/condition, given as a discrete number and unit of measurement
☐	☒	A statement on whether measurements were taken from distinct samples or whether the same sample was measured repeatedly
☐	☒	The statistical test(s) used AND whether they are one- or two-sided<br>*Only common tests should be described solely by name; describe more complex techniques in the Methods section.*
☒	☐	A description of all covariates tested
☐	☒	A description of any assumptions or corrections, such as tests of normality and adjustment for multiple comparisons
☐	☒	A full description of the statistical parameters including central tendency (e.g. means) or other basic estimates (e.g. regression coefficient) AND variation (e.g. standard deviation) or associated estimates of uncertainty (e.g. confidence intervals)
☐	☒	For null hypothesis testing, the test statistic (e.g. *F*, *t*, *r*) with confidence intervals, effect sizes, degrees of freedom and *P* value noted<br>*Give P values as exact values whenever suitable.*
☒	☐	For Bayesian analysis, information on the choice of priors and Markov chain Monte Carlo settings
☒	☐	For hierarchical and complex designs, identification of the appropriate level for tests and full reporting of outcomes
☒	☐	Estimates of effect sizes (e.g. Cohen's *d*, Pearson's *r*), indicating how they were calculated

Our web collection on statistics for biologists contains articles on many of the points above.

Software and code

Policy information about availability of computer code

Data collection	Antibody response data (HI antibodies, MN antibodies, HA total binding antibodies and HA stalk binding bodies) were generated using pre- and post-vaccination sera collected in the randomized trial. Softmax Pro 6.5.1 was used in MN and ELISA data collection.
Data analysis	SAS 9.4 (SAS Institute) and GraphPad Prism 8 (GraphPad Software, Inc.) were used in analyses

For manuscripts utilizing custom algorithms or software that are central to the research but not yet described in published literature, software must be made available to editors and reviewers. We strongly encourage code deposition in a community repository (e.g. GitHub). See the Nature Portfolio guidelines for submitting code & software for further information.

Data

Policy information about availability of data

All manuscripts must include a data availability statement. This statement should provide the following information, where applicable:

- Accession codes, unique identifiers, or web links for publicly available datasets
- A description of any restrictions on data availability
- For clinical datasets or third party data, please ensure that the statement adheres to our policy

The data generated in this study are provided in the Supplementary information/source data file. Sequences of the viruses are available in GISAID (Global Initiative on Sharing Avian Influenza Data"database).

Research involving human participants, their data, or biological material

Policy information about studies with human participants or human data. See also policy information about sex, gender (identity/presentation), and sexual orientation and race, ethnicity and racism.

Reporting on sex and gender	No analysis was performed based on sex and gender
Reporting on race, ethnicity, or other socially relevant groupings	No analysis was performed based on race, ethnicity and other socially relevant data.
Population characteristics	Age is the only characteristics analyzed. Participants (18-64 yrs) were analyzed by 2 age groups: 18-44 years and 45-64 years for their antibody responses following vaccination
Recruitment	Both participants and study investigators were aware of study arm assignments. Laboratory investigators were blinded to assignment until testing was completed. Enrolled HCP stratified by age groups (18–44 years and 45–64 years) were assigned to receive vaccines using a site-stratified REDCap-based randomization system. no self-selection or other biases that may impact results.
Ethics oversight	The study protocol was reviewed and approved by the institutional review boards (IRBs) of the study sites and the Centers for Disease Control and Prevention.

Note that full information on the approval of the study protocol must also be provided in the manuscript.

Field-specific reporting

Please select the one below that is the best fit for your research. If you are not sure, read the appropriate sections before making your selection.

☒ Life sciences ☐ Behavioural & social sciences ☐ Ecological, evolutionary & environmental sciences

For a reference copy of the document with all sections, see nature.com/documents/nr-reporting-summary-flat.pdf

Life sciences study design

All studies must disclose on these points even when the disclosure is negative.

Sample size	The clinical study is a randomized, open-label trial conducted in the United States during the Northern Hemisphere 2018-2019 (Year 1) and 2019-2020 (Year 2) influenza seasons. All samples from the original study with available sera are included in the current analysis. The detailed sample size determination in both study years was described in Dawood FS, et al. Clin Infect Diseases, 2021;73(11):1973–81(ref 27), Gaglani M, et al. Clin Infect Dis, 2022; 76(3): 1168-1176 (Ref 28), and Naleway et al., Open Forum Infectious Diseases 2022; 10(6) (Ref 29) and referenced.
Data exclusions	No data was excluded from analyses.
Replication	For each immunological measurement (HI, MN and ELISA), at least 2 replicates were performed per sample in each assay. The titer variability among the replicates must be within 2 folds otherwise repeat testing is required. Geometric mean titers (GMTs) of at least 2 replicates were reported as final titers for each assay.
Randomization	In year 1, HCP were stratified by two age groups (18-44 years and 45-64 years) and randomized at 4:4:2:2 ratio to receive one of four quadrivalent vaccines: ccIIV4 (Flucelvax), RIV4 (Flublok), Fluzone IIV4 or Fluarix IIV4. In year 2, participants who received ccIIV4, RIV4, and Fluzone IIV4 or Fluarix-IIV4 in Year 1 were re-randomized into seven repeat-vaccination arms: ccIIV4-ccIIV4, ccIIV4-RIV4, RIV4-ccIIV4, RIV4-RIV4, IIV4-ccIIV4, IIV4-RIV4, IIV4-IIV4 (Fluzone), details are described in figure 1. Additional details of the randomization of the original clinical study are described and referenced in: Dawood FS, et al. Clin Infect Diseases, 2021;73(11):1973–81, (ref 27) Gaglani M, et al. Clin Infect Dis, 2022; 76(3): 1168-1176 (Ref 28), and Naleway et al., Open Forum Infectious Diseases 2022; 10(6) (Ref 29). All available samples from the vaccines groups in both year 1 and year 2 were included in the current study.
Blinding	Both participants and study investigators were aware of study arm assignments. Laboratory investigators were blinded to assignment until testing was completed.

Reporting for specific materials, systems and methods

We require information from authors about some types of materials, experimental systems and methods used in many studies. Here, indicate whether each material, system or method listed is relevant to your study. If you are not sure if a list item applies to your research, read the appropriate section before selecting a response.

Materials & experimental systems

n/a	Involved in the study	
☒	☐	Antibodies
☐	☒	Eukaryotic cell lines
☒	☐	Palaeontology and archaeology
☒	☐	Animals and other organisms
☐	☒	Clinical data
☒	☐	Dual use research of concern
☒	☐	Plants

Methods

n/a	Involved in the study	
☒	☐	ChIP-seq
☒	☐	Flow cytometry
☒	☐	MRI-based neuroimaging

Eukaryotic cell lines

Policy information about cell lines and Sex and Gender in Research

Cell line source(s)	MDCK, and MDCK-SIAT1 cells- CDC's International Reagent Resource (IRR)
Authentication	The MDCK-SIAT1 was directly sourced from: Matrosovich et al. J of Virol. 77, 8418-8425 (2003).
Mycoplasma contamination	All cell passages were tested negative for mycoplamsa contamination.
Commonly misidentified lines (See ICLAC register)	No commonly misidentified cell lines were used in this study.

Clinical data

Policy information about clinical studies

All manuscripts should comply with the ICMJE guidelines for publication of clinical research and a completed CONSORT checklist must be included with all submissions.

Clinical trial registration	This study is registered in ClinicalTrials.gov, NCT03722589
Study protocol	study protocol can be accessed at: ClinicalTrials.gov, number NCT03722589
Data collection	The clinical study was a randomized, open-label trial conducted in the United States during the Northern Hemisphere 2018-2019 (Year 1) and 2019-2020 (Year 2) influenza seasons. During Year 1, HCP were stratified by two age groups (18-44 years and 45-64 years) and randomized 4:4:2:2 to receive one of four quadrivalent vaccines: ccIIV4 (Flucelvax), RIV4 (Flublok), IIV4 (Fluzone) or IIV4 (Fluarix) (Figure 1). Serum samples were collected at baseline (day 0), 1-month, and 6-months post-vaccination. The Year 2 study was designed to evaluate the antibody responses following 7 different Year 1-Year 2 repeat vaccination regimens: IIV4-IIV4 (Fluzone), IIV4-ccIIV4, IIV4-RIV4, RIV4-ccIIV4, RIV4-RIV4, ccIIV4-ccIIV4 and ccIIV4-RIV4 (Figure 1). Serum samples were collected at baseline (day 0) and 1-month post-vaccination. The detailed information on clinical data collections are described in: Dawood FS, et al. Clin Infect Diseases, 2021;73(11):1973–81, (ref 27) Gaglani M, et al. Clin Infect Dis, 2022; 76(3): 1168-1176 (Ref 28), and Naleway et al., Open Forum Infectious Diseases 2022; 10(6) (Ref 29).
Outcomes	This study is an exploratory analysis to assess antibody responses to both egg- and cell-propagated vaccine viruses among HCP following vaccination with RIV4, ccIIV4, and standard egg-based IIV4s in two influenza seasons and to investigate whether repeat vaccination with non-egg-based vaccines can overcome the effect of prior repeat vaccination with egg-based vaccines. The primary outcomes of this randomized trial are described in: Dawood FS, et al. Clin Infect Diseases, 2021;73(11):1973–81, (ref 27) Gaglani M, et al. Clin Infect Dis, 2022; 76(3): 1168-1176 (Ref 28), and Naleway et al., Open Forum Infectious Diseases 2022; 10(6) (Ref 29)
